# Acute Intraoperative Saddle Pulmonary Embolus in a Trauma Patient Undergoing Repair of Tibial Fracture: A Case Report

**DOI:** 10.1002/ccr3.71422

**Published:** 2025-11-10

**Authors:** Michael Ayad, Jibran Ikram, A. R. Husnain, Hiba Anis, Sunpreet Tandon, Anokha Padubidri, Sabry Ayad

**Affiliations:** ^1^ Lake Erie College of Osteopathic Medicine Cleveland Ohio USA; ^2^ Outcomes Research Department Cleveland Clinic Cleveland Ohio USA; ^3^ Cleveland Clinic Foundation Cleveland Ohio USA; ^4^ Department of Orthopaedics Cleveland Clinic Fairview Hospital Cleveland Ohio USA; ^5^ Department of Radiology Cleveland Clinic Fairview Hospital Cleveland Ohio USA; ^6^ Cleveland Clinic Fairview Hospital Cleveland Ohio USA

**Keywords:** cannabis use, catheter‐directed thrombectomy, deep vein thrombosis, orthopedic surgery, perioperative care, pulmonary embolism

## Abstract

This case report describes the management of a 40‐year‐old female who developed a saddle pulmonary embolism (PE) following open reduction and internal fixation (ORIF) of a distal tibia fracture. The patient had no significant past medical history except for regular cannabis use. Postoperatively, she developed a deep vein thrombosis (DVT) and a saddle PE, which were managed with catheter‐directed thrombectomy and anticoagulation therapy. This report explores the potential link between cannabis use and thromboembolic events and emphasizes the need for comprehensive perioperative care in patients with such risk factors.

**Summary**

- This case highlights the potential link between cannabis use and an elevated risk of venous thromboembolism (VTE), evidenced by a patient who experienced a saddle pulmonary embolism and deep vein thrombosis following orthopedic surgery.
- Clinicians should include a detailed substance use history in preoperative evaluations to better stratify VTE risk and consider enhanced prophylactic measures. Prompt recognition and management of thromboembolic events are essential for optimizing patient outcomes in high‐risk populations.

## 1 Introduction

Venous thromboembolism (VTE), encompassing deep vein thrombosis (DVT) and pulmonary embolism (PE), is a well‐known complication following orthopedic surgeries, particularly those involving the lower extremities [1, 2]. Common risk factors for VTE include immobility, obesity, smoking, and advanced age. However, the role of cannabis use as a potential contributor to VTE is not well understood. Emerging evidence suggests that cannabis may impact coagulation pathways, thereby increasing the risk of thromboembolic events [3]. In this case report, we present a 40‐year‐old female who developed a saddle PE after distal tibia ORIF, with a focus on the potential association between cannabis use and VTE.

## 2 Case History/Examination

**FIGURE 1:**
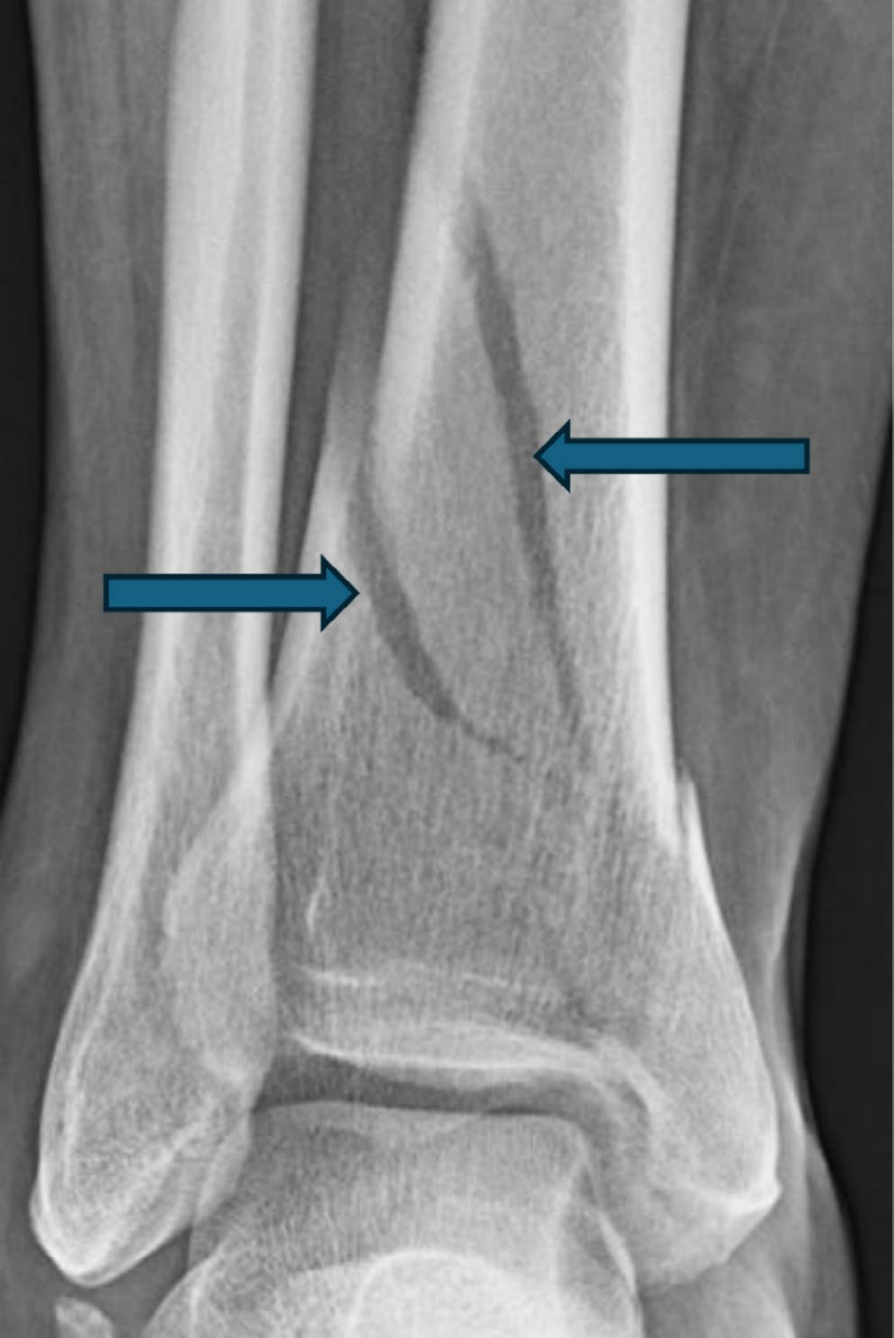
X‐ray showing closed distal tibia fracture.

A 40‐year‐old female presented with a right ankle injury sustained while walking with her dog. Initial examination revealed a closed distal tibia fracture with neurovascular integrity intact (Figure 1). The patient denied the use of oral contraceptives or hormone replacement therapy. She had no prior personal or family history of venous thromboembolism, and no known thrombophilia or clotting disorder. She had no comorbid conditions such as diabetes, hypertension, or malignancy. Importantly, she experienced 10 days of relative immobility prior to surgery while awaiting swelling resolution, Standard DVT prophylaxis with aspirin (81 mg BID) was administered postoperatively.

**FIGURE 2:**
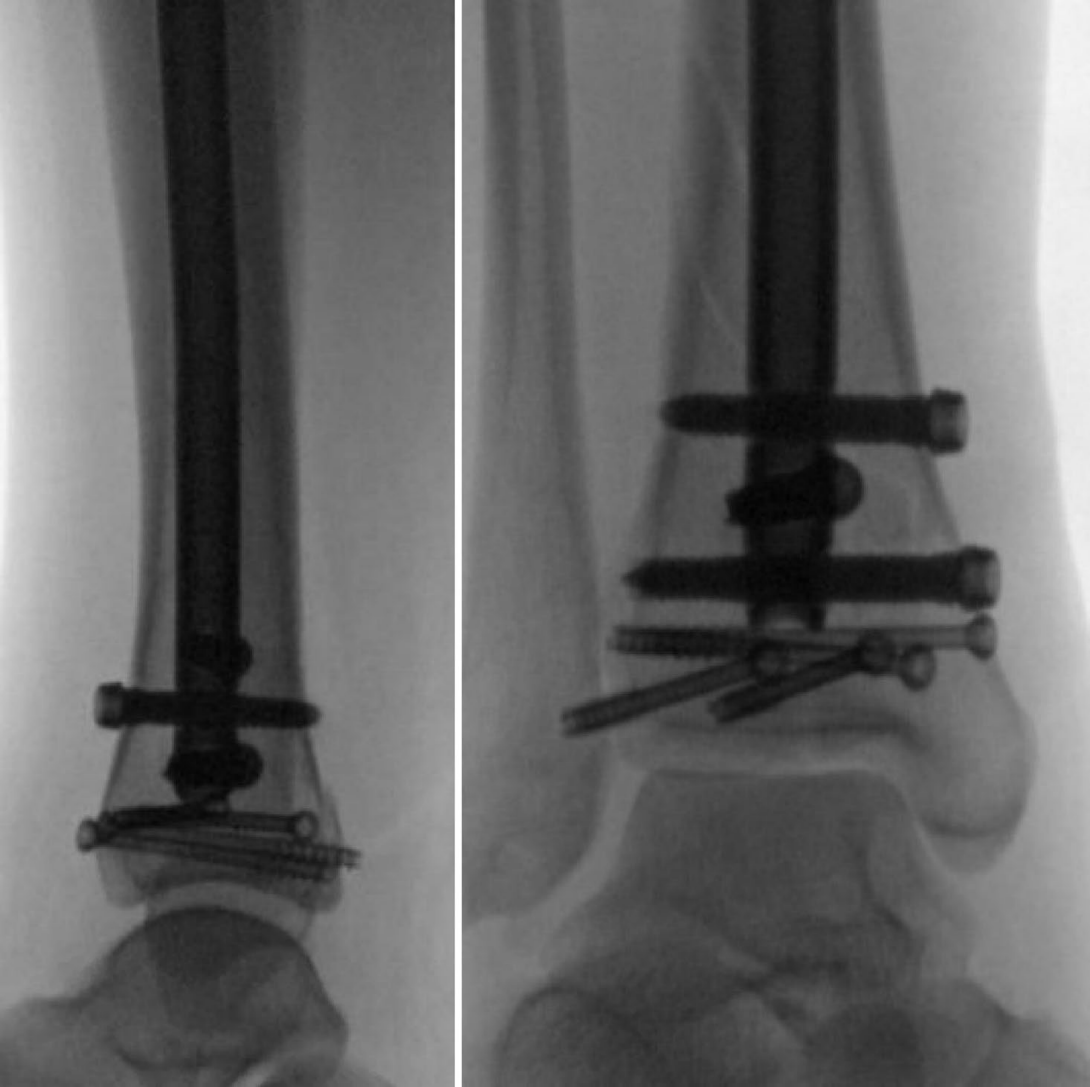
Image showing percutaneous screw fixation of distal tibia followed by suprapatellar intramedullary nail.

The patient underwent open reduction and internal fixation (ORIF) of the distal tibia 10 days post‐injury to allow for swelling to resolve; the risk of infection is higher when ORIF is acutely performed while there is still swelling. The procedure included percutaneous screw fixation of the distal tibia and placement of a suprapatellar intramedullary nail (Figure 2). Postoperatively, she was administered aspirin (81 mg BID) for DVT prophylaxis and received intravenous cefazolin to prevent infection.

**FIGURE 3:**
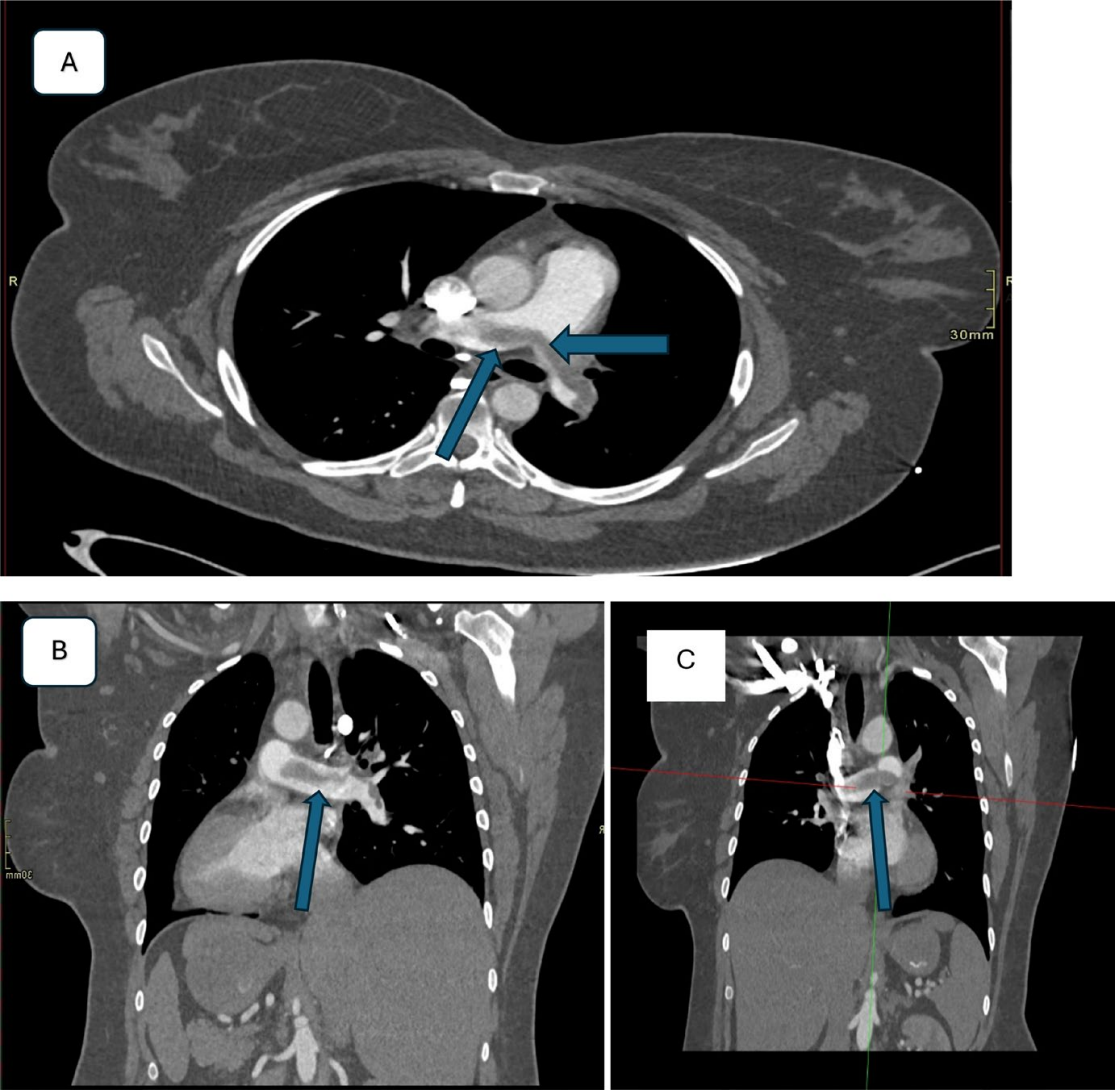
CT pulmonary angiogram showing an acute saddle pulmonary embolus. (A) Axial. (B) and (C) Coronal sections.

**FIGURE 4:**
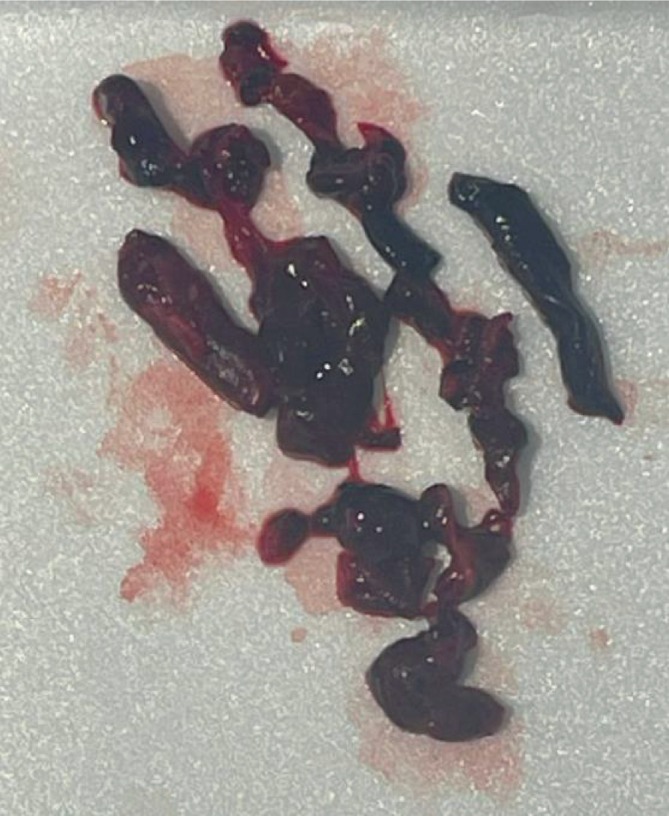
Intraoperative image of pulmonary embolus.

**FIGURE 5:**
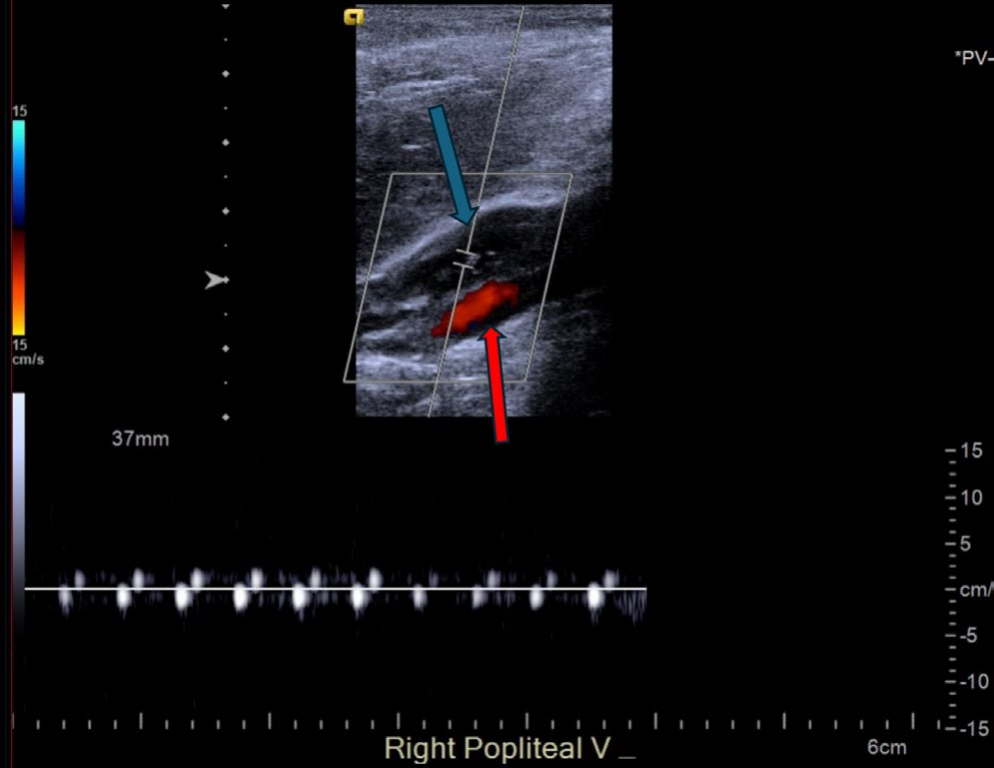
Image showing lower extremity Doppler ultrasound revealing an acute proximal DVT in the right popliteal vein (filling defect). (a) Blue arrow showing popliteal vein. (b) Red arrow showing popliteal artery.

On postoperative day 1, the patient developed chest pain and lightheadedness during mobilization. She exhibited tachycardia, and an EKG showed changes consistent with right heart strain. A CT pulmonary angiogram confirmed an acute saddle PE with right heart strain (Figure 3). She was transferred to the Medical Intensive Care Unit (MICU) and underwent catheter‐directed pulmonary thrombectomy (Figure 4). Bilateral lower extremity Doppler ultrasound revealed an acute proximal DVT in the right popliteal vein (Figure 5). The patient was started on apixaban and discharged after stabilization with instructions for close follow‐up.

At her 2‐week postoperative follow‐up, the patient reported no chest pain or dyspnea. Physical examination showed well‐healed surgical incisions, and Doppler ultrasound confirmed resolution of the DVT. The patient continued apixaban and was advised to remain non‐weight bearing for 6 weeks. At her 6‐week follow‐up, she was progressed to weight‐bearing as tolerated and continued physical therapy.

## 3 Methods (Differential Diagnosis, Investigations and Treatment)

Upon presentation with acute chest pain, tachycardia, and lightheadedness following her distal tibia ORIF, the patient underwent a series of targeted investigations to determine the etiology of her symptoms. An initial electrocardiogram (EKG) demonstrated findings suggestive of right ventricular strain, raising clinical suspicion for a pulmonary embolism (PE). This prompted a CT pulmonary angiography (CTPA), which confirmed a saddle PE with associated right heart strain. To further assess for thromboembolic sources, bilateral lower extremity Doppler ultrasound was performed, identifying an acute proximal deep vein thrombosis (DVT) in the right popliteal vein. These diagnostic findings substantiated a diagnosis of postoperative thromboembolic events and guided immediate management. The patient underwent catheter‐directed thrombectomy to relieve the obstructive thrombus burden, followed by therapeutic anticoagulation with apixaban to mitigate recurrence risk.

## 4 Conclusion and Result (Outcomes and Follow‐Up)

The case underscores the need for heightened awareness of potential thromboembolic risk factors, including cannabis use, in orthopedic patients. The patient experienced favorable outcomes following catheter‐directed thrombectomy and anticoagulation therapy, evidenced by the resolution of her pulmonary embolism and deep vein thrombosis. During follow‐up, the patient reported no residual symptoms, and imaging confirmed DVT resolution, supporting the effectiveness of her treatment plan. Continued use of apixaban and a structured rehabilitation plan facilitated her full recovery, allowing her to progress to weight‐bearing activities and resume normal function. Further research is warranted to clarify the relationship between cannabis use and VTE and to inform guidelines for perioperative management in this patient population.

## 5 Discussion

This case highlights the challenges of managing thromboembolic complications following orthopedic surgery, particularly in the context of cannabis use. Cannabis has been increasingly recognized as a substance that may influence hemostasis, with studies suggesting that cannabinoids can affect platelet function and the endocannabinoid system, potentially increasing the risk of thrombus formation [4].

Cannabinoid receptors, primarily CB1 and CB2, are expressed in various tissues, including the vascular endothelium and platelets. Activation of these receptors can lead to altered platelet aggregation and vasodilation, which may contribute to an increased risk of thromboembolic events [5]. Furthermore, chronic cannabis use has been associated with an increased incidence of cardiovascular events, including myocardial infarction and stroke, which share common pathophysiological mechanisms with VTE [3].

In this patient, the development of a saddle PE and DVT shortly after surgery raises questions about the potential role of cannabis as a contributing factor. While her regular use of cannabis may not have been the sole cause of the thromboembolic event, it likely compounded other risk factors associated with orthopedic surgery, such as immobility and tissue injury. The management of her PE through catheter‐directed thrombectomy, followed by anticoagulation with apixaban, was successful, underscoring the importance of timely and aggressive treatment in such cases.

Recent large‐scale studies have not demonstrated a statistically significant association between cannabis use and venous thromboembolism. These findings suggest that cannabis alone may not be an independent risk factor for VTE. However, in our case, chronic cannabis use may have acted synergistically with strong perioperative risk factors such as immobility and orthopedic surgery to potentiate thrombus formation. Thus, while cannabis use may not universally predispose to VTE, it remains an important consideration in comprehensive perioperative risk stratification [6, 7].

Given the potential for cannabis to influence coagulation, it is crucial for clinicians to obtain a thorough substance use history as part of the preoperative assessment. This information could guide the use of more aggressive DVT prophylaxis strategies in patients at higher risk. Additionally, the growing prevalence of cannabis use, particularly in regions where it is legalized, suggests that clinicians may encounter more patients with similar risk profiles. This underscores the need for further research to better understand the relationship between cannabis use and VTE, and to inform guidelines on perioperative care in this population.

## Author Contributions

**Michael Ayad:** data curation, formal analysis, investigation, methodology, project administration, software, validation, visualization, writing – original draft, writing – review and editing. **Jibran Ikram:** conceptualization, data curation, formal analysis, investigation, methodology, project administration, resources, software, validation, visualization, writing – original draft, writing – review and editing. **A.R Husnain:** resources, software, validation, writing – review and editing. **Hiba Anis:** conceptualization, investigation, methodology, project administration, resources, software, validation, visualization, writing – original draft, writing – review and editing. **Sunpreet Tandon:** conceptualization, data curation, formal analysis, funding acquisition, investigation, methodology, project administration, software, validation, visualization, writing – original draft, writing – review and editing. **Anokha Padubidri:** conceptualization, data curation, formal analysis, funding acquisition, investigation, methodology, project administration, resources, software, supervision, validation, visualization, writing – review and editing. **Sabry Ayad:** conceptualization, data curation, formal analysis, funding acquisition, investigation, methodology, project administration, resources, software, supervision, validation, visualization, writing – original draft, writing – review and editing.

## Consent

Written informed consent was acquired from the patient whose clinical images and case details are written in the study, to publish this report in accordance with the journal's patient consent policy.

## Conflicts of Interest

The authors declare no conflicts of interest.

## Data Availability

The data supporting this report's findings are available on request from the corresponding author. The data is not publicly available due to privacy or ethical restrictions.
